# Hepatocellular Carcinoma among Patients with Chronic Liver Disease in a Tertiary Care Centre: A Descriptive Cross-sectional Study

**DOI:** 10.31729/jnma.8488

**Published:** 2024-03-31

**Authors:** Arun Gnawali, Rahul Pathak, Dinesh Koirala, Rajesh Pandey, Rabin Hamal, Anurag Jha, Brindeswari Kafle Bhandari, Siddinath Gyawali

**Affiliations:** 1 Department of Gastroenterology, Tribhuvan University Teaching Hospital, Institute of Medicine, Maharajgunj, Kathmandu, Nepal; 2 Maharajgunj Medical Campus, Institute of Medicine, Maharajgunj, Kathmandu, Nepal

**Keywords:** *autoimmune hepatitis*, *hepatitis*, *hepatocellular carcinoma*, *treatment*

## Abstract

**Introduction:**

Hepatocellular carcinoma is the most common primary liver cancer. Viral hepatitis, alcohol abuse, and autoimmune hepatitis are the common causes of hepatocellular carcinoma. Usually patients present at advanced stages where curative treatment is no longer possible. This study aimed to find the prevalence of hepatocellular carcinoma among patients with chronic liver disease in a tertiary care centre.

**Methods:**

This is a descriptive cross-sectional study conducted in a single tertiary care centre from March 2020 to August 2022. The study was done among inpatients of the Department of Gastroenterology after ethical approval from the Institutional Review Committee. Convenience sampling method was used and data were collected using predetermined proformas. Point estimate at 95% Confidence Interval was calculated.

**Results:**

Among 1440 patients, hepatocellular carcinoma was seen in 54 (3.75%) (2.77-4.73, 95% Confidence Interval). At the time of diagnosis, 48 (88.89%) were symptomatic. The presenting symptoms were weight loss seen in 35 (64.81%) being the most common. Out of them, 37 (68.52%) consumed alcohol and 40 (74.07%) smoked cigarettes.

**Conclusions:**

Hepatocellular carcinoma is a notable concern. Alcohol-related liver cirrhosis is the most frequent condition encountered in patients with hepatocellular carcinoma in our setting.

## INTRODUCTION

Liver cancer is one of the most common cancers worldwide which occupies fifth and seventh ranks in terms of incidence among males and females respectively, and fourth position in overall mortality.^1^ Due to lower prevalence rates of hepatitis B virus (HBV) and hepatitis C virus (HCV) infections in Nepal, which are 0.9% and 0.4% respectively, Nepal has a low incidence of hepatocellular carcinoma (HCC).^2^

According to the National Cancer Registry of Nepal (2013), the incidence of liver cancer in Nepal in men and women is 0.8 and 0.9 per 100,000 population respectively.^3^ Though the cancer registry began in Nepal in 2003, the registry is not comprehensive, and there are very limited studies conducted on liver cancer in Nepal.^2^

The study aimed to find the prevalence of hepatocellular carcinoma among patients with chronic liver disease admitted to the Department of Gastroenterology of a tertiary care centre.

## METHODS

This is a descriptive cross-sectional study conducted from March 2020 to August 2022 among patients admitted to the ward of the Department of Gastroenterology of Tribhuvan University Teaching Hospital. The data collection was done prospectively after the study was approved by the Institutional Review Committee of the Institute of Medicine (Reference number: 132/(6-11) E2/076/077). The patients with chronic liver disease admitted to the ward consenting to the study were included in this study. The patients with secondary liver cancer and those with incomplete medical records were excluded from the study. A convenience sampling method was used for taking the study sample. The sample size was calculated using the following formula:


$$\begin{array}{ll} \text{n} & ={\text{Z}}^{2}\times \frac{\text{p}\times \text{q}}{{\text{e}}^{\text{2}}}\\ & =1.96^{2}\times \frac{0.50\times 0.50}{0.03^{2}}\\ & =1041 \end{array}$$

Where,

n = minimum required sample size
Z = 1.96 at 95% Confidence Interval (CI)
p = prevalence taken as 50% for maximum sample size
q = 1-p
e = margin of error, 3%

The calculated sample size was 1041. All the patients in between the study period were taken and inclusion and exclusion criteria were applied.

Cirrhosis was diagnosed by the combination of clinical features of chronic liver disease, laboratory reports of decreased liver synthetic and excretory functions, and imaging features of irregular liver outline, dilated portal vein, and collaterals combined with endoscopic screening of esophageal varices.^4^ The diagnosis of HCC was made by detailed clinical examination, and imaging modality (dynamic CT scan or MRI of the abdomen) which shows early arterial enhancement and venous washout of the lesion, or with the biopsy of the lesion whenever necessary.^5^ Child-Turcotte-Pugh (CTP) status was calculated in all patients. Staging of HCC was done using Barcelona Clinic Liver Cancer Staging.^6^

The risk factors, clinical characteristics, and treatment modalities in patients with HCC were thoroughly assessed. Predetermined proforma was used as a tool for data collection. The data were tabulated in Microsoft Excel 2016 and statistical analysis was done using statistical software. Results on continuous variables were expressed as Mean±Standard deviation (SD) and results on categorical variables were expressed in number and percentages. The demographic profile of the patients, aetiology, clinical presentation, malignancy characteristics, and management modalities have been expressed in numbers and frequencies, whereas laboratory parameters have been demonstrated in Mean±SD. Point estimate at 95% Confidence Interval (CI) was calculated.

## RESULTS

In this study, hepatocellular carcinoma was found among 54 (3.75%) (2.77-4.73, 95% CI). The youngest patient in our study was 28 years old, the oldest was 81 years, and the mean age was 57.17 years. Of them, 46 (85.18%) were males and 8 (14.82%) were females. Among total, 37 (68.52%) consumed alcohol and 40 (74.07%) were smokers.Comorbidity in the form of diabetes mellitus was present in 5 (9.26%), hypertension in 6 (14.81%), and both diabetes and hypertension were present in 8 (11.11%) patients (Table 1).

**Table 1:** Demographic profile of the patients with HCC (n = 54).

Patient characteristics	n (%)
**Age wise distribution (in years)**	
≤40	6 (11.11)
40-49	8 (14.81)
50-59	14 (25.92)
60-69	13 (24.07)
≥70	13 (24.07)
**Gender**	
Male	46 (85.18)
Female	8 (14.82)
**HCC diagnosis**	
Symptomatic	48 (88.89)
Incidental	6 (11.11)
**Comorbidities**	
Diabetes mellitus	8 (14.81)
Hypertension	5 (9.26)
Hypertension and diabetes mellitus	6 (11.11)
Alcohol use	37 (68.52)
Smoking	40 (74.07)

Alcohol abuse found in 21 (38.89%) cases followed by chronic viral hepatitis B (CHB) infection in 6 (11.11%). In addition, 12 patients (22.22%) had a history of both alcohol consumption as well as hepatitis B infection (Table 2).

**Table 2:** Underlying factors in the patients with HCC (n = 54).

Factors	n (%)
Alcohol	21 (38.89)
Alcohol + Hepatitis B infection	12 (22.22)
Hepatitis B infection	6 (11.11)
Cryptogenic	6 (11.11)
Alcohol + hepatitis C infection	4 (7.41)
Hereditary hemochromatosis	3 (5.56)
Non-alcoholic fatty liver disease (NAFLD)	2 (37.03)

At the time of diagnosis, 48 (88.89%) were symptomatic. The presenting symptoms were weight loss seen in 35 (64.81%), abdominal distension in 33 (61.11%), anorexia in 33 (61.11%), and abdominal pain in 27 (50.0%). Ascites was present in 32 (59.26%), which were further graded as I in 5 (15.63%), grade II in 4 (12.50%) and grade III in 23 (71.88%). Hepatic encephalopathy was found in only 4 (7.41%) patients.

All patients (100%) underwent imaging by either a triple phase computed tomography (CT) scan or an MRI of the abdomen. The HCC involved the right lobe of liver in 30 (55.56%) followed by the bilobar involvement in 16 (29.63%). The only left lobe involvement was found in 8 (14.78%) patients. Multicentric HCC with more than three lesions was the radiological pattern seen in 19 (35.19%) followed by the single lesion seen in 15 (27.78%). Vascular involvement of the porto-splenic axis was seen in 24 (44.44%) patients. Extrahepatic spread of the tumour was seen in 8 (14.81%) patients. Regional periportal lymph node involvement was seen in 4 (7.41%), 2 (3.70%) had metastasis to the lung, and the remaining 2 (3.70%) had metastasis to the peritoneum. On detailed evaluation, 42 (77.78%) patients were found to have underlying liver cirrhosis (Table 3).

**Table 3:** Malignancy characteristics of HCC (n = 54).

Malignancy characteristics	n (%)
**No. of lesion**	
1	15 (27.78)
2	8 (14.81)
3	12 (22.22)
>3	19 (35.19)
**Lobe of liver**	
Right	30 (55.56)
Left	8 (14.81)
Both	16 (29.63)
**CTP status**	
A	23 (42.59)
B	14 (25.93)
C	17 (31.48)
**ECOG performance status**	
0	20 (37.04)
1	8 (14.81)
2	11 (20.37)
3	11 (20.37)
4	4 (7.41)
**BCLC Staging**	
0	-
A	8 (14.81)
B	6 (11.11)
C	19 (35.19)
D	21 (38.89)
**Distant metastasis**	
Yes	8 (14.81)
No	46 (85.19)
**Portal vein thrombosis**	
Yes	24 (44.44)
No	30 (55.56)
**Acute kidney injury**	
Yes	8 (18.81)
No	46 (85.19)
**AFP elevated**	
Yes	44 (81.48)
No	10 (18.52)
**Liver status**	
Cirrhotic Decompensated	34 (62.97)
Compensated	8 (14.81)
Non-cirrhotic	12 (22.22)

The evaluation of laboratory parameters among the participants demonstrated that the serum alphafetoprotein was elevated in 44 (81.48%) patients with a mean value of 525±37 ng/ml. The mean values of CTP score, Model for End-Stage Liver Disease (MELD) score, and MELD-Na score were calculated (Table 4).

**Table 4:** Laboratory parameters of patients with HCC (n = 54).

Parameters	Mean±SD
Haemoglobin (g/dl)	10.90±2.70
Total bilirubin (micromole/l)	60.60±13
Serum albumin (g/l)	32.20±4
Sodium (meq/l)	135±4
AST (IU/L)	114±20
ALT (IU/L)	52±11
ALP (IU/L)	525±44
AFP level (ng/ml)	525±37
CTP score	7.70±1.60
MELD score	12.78±3
MELD-Na score	14.19±4.20

A total of 50 (92.59%) underwent non-surgical treatment and only 4 (7.41%) patients underwent surgical treatment i.e., tumour resection. Among non-surgical treatment modalities, only 2 (3.70%) patients underwent therapy with curative intent in the form of microwave ablation. The transarterial chemoembolization (TACE) was done in 10 (18.50%) patients and chemotherapy with sorafenib in 17 (31.48%) as palliative therapeutic measures (Table 5).

**Table 5:** Management modalities with HCC (n = 54).

Management Modalities	n (%)
Surgical	4 (7.41)
Non-surgical	50 (92.59)
Potentially curative (micro-ablation)	2 (3.70)
Palliative TACE	10 (18.52)
Sorafenib	17 (31.48)
Best supportive	21 (38.89)

## DISCUSSION

The study showed a prevalence of hepatocellular carcinoma to be 3.75% among chronic liver disease cases done in a single centre of Nepal. This is the largest study in Nepal of this type where we studied the clinical, etiological, and radiological profile of 54 patients with HCC admitted to the ward under the Department of Gastroenterology.

Published studies have shown that the incidence of HCC increases with age such that its occurrence before 40 years of age is minimal in the Western world.^4^ The age distribution of patients with HCC in this study is similar to a study by Kumar R. et al. in India which has shown the maximum incidence of HCC in the sixth decade.^7^ Regarding the sex-wise distribution of HCC, our study showed that there is a male preponderance in the prevalence of HCC in our study with a male:female ratio of 5:1. Similar finding with a higher preponderance of HCC in male is shown in other previous studies with the male: female ratios ranging from 3:1 to 10:1.2,^7,8^ This suggests that older age and male sex are the risk factors for liver cancer. However, the higher incidence of HCC in males might be because males are more likely to drink alcohol and smoke cigarettes.^9^ Recent studies have shown that oestrogen in females may protect them from HCC as compared to males.^10^

The current study demonstrated that most of the patients 88.89% were symptomatic at the time of presentation, and the most common presentations were abdominal discomfort/pain, abdominal distention, and anorexia. This finding corresponds with other similar studies where only 16.81% of total patients were asymptomatic and the rest presented with similar complaints.^8,11^ This reflects the late presentation of the patients with HCC to the medical facility when the tumour is no longer resectable with complaints like abdominal pain and abdominal distension.

Non-cirrhotic HCC accounted for 22.22% of all patients in our study, while the majority 77.78% had liver cirrhosis. Most of the patients with cirrhosis 80.95% had decompensated disease in various forms. A multinational cross-sectional study by Yang J.D et al. in Africa revealed that all the patients with HCC in Egypt (100%) and around two-thirds in other African nations (66%) had cirrhosis. The findings in our study and other similar studies suggest that hepatic cirrhosis is also a strong risk factor for HCC.^12^

The etiological risk factors for HCC vary in different geographical regions. The majority of our patients with HCC had alcohol abuse as a cause of cirrhosis, and HBV as the sole cause of cirrhosis was seen only in 11% of patients. These findings contradict the outputs of other studies conducted in India which found HBV as the most common cause of HCC.^7,11^ However, a similar study conducted by Egypt showed HBV infection in only 22.4% of total cases of HCC.^13^ Similarly, another study by Aljumah A. et al. indicated HCV infection as the most common cause of HCC affecting 46.80% of the total cases.^14^ This could be due to the fact that there is a very low prevalence of HBV and HCV in Nepal, and alcohol-related cirrhosis is more common in our country.^15-17^ Serum AFP level was elevated in 81.48% of our patients and median AFP level was 286 ng/ml. In a study by Bhatti et al., the median AFP level among patients with HCC was only 43.6 ng/ml. This difference could be because most of the patients presented with advanced HCC in our study. Though serum AFP measurement is used as a screening tool for HCC, the rise in its levels in serum is neither sensitive nor specific to HCC.^18, 19^

Most of our patients presented either with advanced stage (BCLC stage C) (35.19%) or terminal stage (BCLC stage D) (38.89%) HCC despite the fact that the majority (42.59%) of them had Child-Turcotte-Pugh (CTP) score A. This is in contrast to the study by Aljumah A. et al. where BCLC combined stages A and B comprise nearly 70% of total cases, with the majority having preserved liver function, CTP score class A.^14^ Moreover, macrovascular invasion and metastases were seen in 44.44% and 14.81% of patients respectively, which is in accordance with the study, by Masunuri et al., where portal vein thrombosis (PVT) was seen in 40.4% of patients.^5^ However other similar studies found PVT in less than 20% of total cases only.^13, 14^ Since, tumour cells are more likely to disseminate via portal circulation to distant organs, the presence of PVT in HCC is a critical issue leading to early deterioration of hepatic function and worse prognosis.^14^ Thus, PVT was the major reason that more than one-third of the patients in our study belonged to the advanced stage HCC despite being in a good functional status. Notably, potentially curative therapies were underutilised i.e. only 7.41% received surgical and 3.70% received non-surgical curative services in the form of microwave ablation. Because of the presence of poor prognostic factors and aggressive behaviour of HCC, 38.89% of them received only the best supportive care for their symptoms. However, the treatment approaches for patients with HCC in Africa varied compared to our setting. As shown in previous studies, 35% of total HCC patients in Egypt and less than 1% in other African countries received curative treatment.^12^

In this study, there are certain limitations. Being a single-centre study, it does not reflect the actual scenario of HCC in Nepal and therefore the results are not generalizable. Our study had a limited number of patients. We were unable to detect whether the noncirrhotic HCC patients had underlying fibrosis or not. Being a tertiary care centre, most cases presented at advanced stages, therefore, we could not follow up on the actual outcome of the treatment in all the stages.

## CONCLUSIONS

Our study depicted HCC was more prevalent in older age, male gender, excess alcohol intake, viral hepatitis, and liver cirrhosis in our setting. Since most patients are asymptomatic in the initial stage of cancer, it is already late when they become symptomatic and present to us when curative treatment is no longer possible. Available curative treatment is still underutilised. We need to strengthen screening of the of HCC in known cirrhotics so that the cases can be detected early when curative treatment is still possible. Being a singlecentre study, the findings are not generalizable to all the Nepalese population. Therefore, we suggest a multicenter study to draw a clearer picture of the prevalence, risk factors, clinical presentation, and treatment modalities of HCC in Nepal.
